# Supplementary material for: An Abattoir-Based Study on the Prevalence of Salmonella Fecal Carriage and ESBL Related Antimicrobial Resistance from Culled Adult Dairy Cows in Wuhan, China
Source: Pathogens. 2020 Oct 19;9(10):853. doi: 10.3390/pathogens9100853 (PMC7590148; doi:10.3390/pathogens9100853)
Supplement: Supplementary file 1 [file pathogens-09-00853-s001.pdf]

Table S1. Information of *Salmonella* isolates in the abattoir.

No.	Distribution	Age (Year)	Serotype	ESBL Phenotype	ESBLs Gene	FFC	PB	CEF	SXT	AMP	ENR	TET	IPM	GEN
1	Central China	> 5	Could not be typed	No	Could not be typed	S	S	S	I	S	S	R	S	S
2	North China	> 5	Could not be typed	No	Could not be typed	S	S	I	I	S	S	S	S	S
3	Central China	> 5	Could not be typed	No	Could not be typed	R	S	S	I	S	I	R	S	S
4	Central China	2	Typhimurium	No	Could not be typed	S	S	S	S	R	S	S	S	R
5	Central China	> 5	Typhimurium	No	Could not be typed	S	S	S	S	R	S	S	S	S
6	North China	> 5	Typhimurium	No	Could not be typed	S	S	S	I	R	I	S	S	S
7	North China	> 5	Typhimurium	No	Could not be typed	R	S	S	R	R	R	R	S	R
8	North China	4	Typhimurium	No	Could not be typed	R	S	S	I	R	R	R	S	R
9	North China	> 5	Typhimurium	No	Could not be typed	R	S	S	I	R	R	R	S	R
10	North China	> 5	Typhimurium	No	Could not be typed	S	S	S	S	R	S	S	S	S
11	North China	3	Could not be typed	No	Could not be typed	R	S	S	R	R	I	R	S	R
12	Northeast China	4	Typhimurium	No	Could not be typed	R	S	S	I	R	R	R	S	R
13	North China	4	Typhimurium	No	Could not be typed	S	S	S	S	R	S	S	S	S
14	North China	> 5	Typhimurium	No	Could not be typed	R	S	R	I	R	R	R	S	R
15	lack	4	Typhimurium	Yes	CTX-M	S	S	R	I	R	S	R	S	S

16	Northeast China	> 5	Typhimurium	No	Could not be typed	S	S	I	S	R	S	S	S	S
17	Northeast China	> 5	Could not be typed	No	Could not be typed	R	S	S	S	R	R	R	S	S
18	Northeast China	> 5	Could not be typed	No	Could not be typed	R	S	S	S	R	I	R	S	S
19	Central China	> 5	Could not be typed	No	Could not be typed	R	S	S	S	R	I	R	S	S
20	Northeast China	> 5	Could not be typed	No	Could not be typed	R	S	S	I	R	R	R	S	S
21	Northeast China	> 5	Typhimurium	No	Could not be typed	S	S	R	S	R	S	S	S	S
22	Northeast China	> 5	Could not be typed	No	CTX-M	S	S	R	I	R	R	R	S	R
23	Northeast China	> 5	Typhimurium	No	Could not be typed	S	S	R	S	R	S	S	S	S
24	Northeast China	> 5	Typhimurium	Yes	CTX-M	S	S	R	I	R	S	R	S	S
25	Northeast China	> 5	Typhimurium	No	Could not be typed	S	S	S	S	R	S	S	S	S
26	Northeast China	> 5	Typhimurium	No	Could not be typed	S	S	S	S	R	S	R	S	S
27	Northeast China	> 5	Typhimurium	No	Could not be typed	S	S	R	S	R	S	R	S	S
28	North China	> 5	Could not be typed	Yes	CTX-M	S	S	R	S	R	R	R	S	R
29	North China	> 5	Could not be typed	Yes	CTX-M	R	S	R	I	R	R	R	S	R
30	Northeast China	> 5	Could not be typed	No	CTX-M	S	S	R	I	R	R	R	S	R
31	Northeast China	> 5	Typhimurium	No	Could not be typed	S	S	R	I	R	S	S	S	S
32	Northeast China	4	Typhimurium	No	Could not be typed	S	S	I	S	R	S	S	S	S

33	Northeast China	> 5	Typhimurium	No	Could not be typed	S	S	R	S	R	S	S	S	S
34	Northeast China	> 5	Typhimurium	Yes	CTX-M	S	S	R	S	R	S	R	S	S
35	Northeast China	> 5	Typhimurium	Yes	CTX-M	S	S	R	I	R	S	R	S	S
36	North China	3	Typhimurium	No	Could not be typed	S	S	S	S	R	S	S	S	S
37	North China	> 5	Typhimurium	No	Could not be typed	S	S	S	S	R	S	S	S	S
38*	North China	> 5	Could not be typed	No	Could not be typed	R	S	R	S	S	S	R	S	S
39*	North China	> 5	Could not be typed	Yes	CTX-M	S	S	R	S	R	S	S	S	S
40	North China	4	Typhimurium	No	Could not be typed	S	S	S	S	R	S	S	S	S
41	Northeast China	3	Dublin	No	Could not be typed	S	S	S	S	S	S	S	S	S

FFC: Florfenicol; PB: Polymyxin B; CEF: Ceftiofur; SXT: Sulfamethoxazole-Trimethoprim; AMP: Ampicillin; ENR: Enrofloxacin; TET: Tetracycline; IPM: Imipenem; GEN: Gentamicin; S: sensitivity; R: resistance; I: intermediary. * From the same sample.

Table S2. The combination of multi-drug resistance.

Combination	Number of Isolates
SXT+GEN+ENR+TET+AMP+FFC	1
SXT+GEN+TET+AMP+FFC	1
GEN+ENR+TET+AMP+FFC+CEF	2
GEN+ENR+TET+AMP+FFC	3
ENR+TET+AMP+FFC	2
GEN+ENR+TET+AMP+CEF	3
TET+AMP+CEF	5
TET+FFC+CEF	1
TET+AMP+FFC	2

SXT: Sulfamethoxazole-Trimethoprim; GEN: Gentamicin; ENR: Enrofloxacin; TET: Tetracycline; AMP: Ampicillin; FFC: Florfenicol; CEF: Ceftiofur.
